# Surgical Management of a Large Multiloculated Thoracolumbar Spinal Extradural Arachnoid Cyst via Decompressive Laminectomy With Selective Foraminal Fenestration: A Case Report

**DOI:** 10.7759/cureus.111332

**Published:** 2026-06-22

**Authors:** Afra S Al-Suhaymi, Nasseer M Sayed

**Affiliations:** 1 Neurosurgery, King Salman Specialist Hospital, Hail, SAU

**Keywords:** cerebrospinal fluid leak, cyst fenestration, laminectomy, nabors type ia, spinal cord compression, spinal extradural arachnoid cyst, thoracolumbar spine

## Abstract

Spinal extradural arachnoid cysts (SEACs) are rare, benign, non-neoplastic lesions arising from focal dural defects, most frequently reported in the thoracic spine. Large multilocular with intraforaminal extension SEACs represent a challenging surgical case, as attempts for complete resection may result in dural defect and cerebrospinal fluid (CSF) leakage. The optimal surgical approach in the setting of adherent foraminal involvement remains an area of debate. We report a 32-year-old woman who presented with a three-month history of progressive low back pain, bilateral lower limb pain and weakness, paresthesia, and intermittent urinary incontinence. Magnetic resonance imaging (MRI) demonstrated a large, lobulated, extradural arachnoid cyst extending across the thoracolumbar junction from lower T10 to upper L2, measuring approximately 116 × 61 × 21 mm, with marked anterior displacement of the spinal cord and lateral foraminal extension in the left T11/T12, T12/L1, and L1/L2 levels. The patient underwent partial laminectomy of the inferior half of T10, complete laminectomy of T11 to T12, and partial laminectomy of the superior half of L1 with near-total cyst resection. The adherent intraforaminal component at the left T11/T12 level was selectively fenestrated to avoid the risk of postoperative dural injury, CSF leakage, or injury to the nerve root. Histopathological examination was consistent with a benign arachnoid cyst, negative for malignancy. Postoperative MRI confirmed complete resolution of all extradural cystic lesions at 10-month follow-up. Pain scores were obtained preoperatively and postoperatively from the patient using the 0-10 numeric rating scale, improved from 9/10 preoperatively to 4/10 at 10-month follow-up; paresthesia and urinary incontinence were reported as fully resolved.

## Introduction

Spinal extradural arachnoid cysts (SEACs) are rare, benign, non-neoplastic lesions resulting from focal defects in the spinal dura mater, through which the arachnoid membrane herniates to form an extradural CSF-filled sac. SEACs are most frequently reported in the thoracic and thoracolumbar spine [1-4]. Using the widely accepted Nabors classification of spinal meningeal cysts, SEACs without nerve root fibers are classified as type IA, whereas type II cysts contain nerve root fibers [1].

The etiology of SEACs remains incompletely understood. Proposed mechanisms include congenital meningeal dysplasia with arachnoid herniation through a developmental dural defect, acquired dural injury secondary to trauma or chronic inflammation, and a ball-valve mechanism in which pulsatile CSF dynamics progressively enlarge the cyst over time [5-8]. Clinically, the spectrum of presentation ranges from incidental asymptomatic discovery to progressive myelopathy, radiculopathy, and sphincter dysfunction - manifestations generally related to the degree and duration of neural compression [1,4,6-8].

The treatment of symptomatic SEACs is surgical. Operative strategies include complete cyst excision, fenestration or marsupialization into the subarachnoid space, and shunt placement or aspiration. Complete resection with dural defect repair is a traditional surgical approach when safely feasible; however, when cyst walls are densely adherent to neural foraminal structures or the dural sleeve, complete excision carries a substantial risk of CSF fistula formation, dural injury, neural injury, and postoperative deformity or instability [7-10]. Selective fenestration of adherent components and closure of dural defect in such cases may represent a safe operative strategy with lower future complications risk, especially in large-sized extradural arachnoid cyst [7,9,11,12].

We describe a case of a young woman with a large thoracolumbar SEAC complicated by multilocularity and adherent intra-foraminal extension, illustrating a systematic surgical approach that prioritized complete neural decompression while using a safe approach to avoid possible complications that may be caused by attempts to resect loculated cysts that are densely adherent with intra-foraminal extension, and reporting the histopathological confirmation as well as functional outcomes.

Informed consent for treatment and open access publication was obtained or waived by all participants in this study.

## Case presentation

A 32-year-old woman with no significant past medical or surgical history presented to the neurosurgery outpatient clinic with a three-month history of progressive low back pain, bilateral lower extremity pain and weakness, subjective paresthesia, and intermittent urinary incontinence. She reported limitations of her daily activities due to lower-limb pain, with minimal response to regular analgesic therapy recently.

On physical examination, the patient was alert, oriented, and hemodynamically stable, with a Glasgow Coma Scale (GCS) score of 15/15. Neurological assessment revealed mild bilateral lower extremity weakness, graded 4/5. Upper extremity power was intact at 5/5 bilaterally. Sensory examination, coordination testing, and deep tendon reflexes were preserved. The remainder of the systemic examination was unremarkable.

Radiological findings

Preoperative MRI of the dorsolumbar spine demonstrated a large, well-defined, lobulated, extra-axial extradural cystic lesion exhibiting low signal intensity on T1-weighted images and uniformly high signal intensity on T2-weighted and STIR sequences, without appreciable post-contrast enhancement (Figures 1-3). The lesion measured approximately 116 × 61 × 21 mm (craniocaudal × transverse × anteroposterior), measured on coronal, axial, and sagittal T2-weighted images, respectively, extending across the thoracolumbar junction from lower T10 to upper L2. Significant anterior displacement of the thoracolumbar cord and adjacent nerve roots was found. Lateral extension into the neural exit foramina was documented at left T11/T12, T12/L1, and L1/L2 levels, with corresponding foraminal widening. Posterior vertebral body scalloping and remodeling of the posterior spinal elements at the involved levels were identified, consistent with a chronic process. The conus medullaris and cauda equina appeared morphologically normal. The overall imaging features were most suggestive of a spinal extradural arachnoid cyst (Nabors type IA).

**Figure 1:**
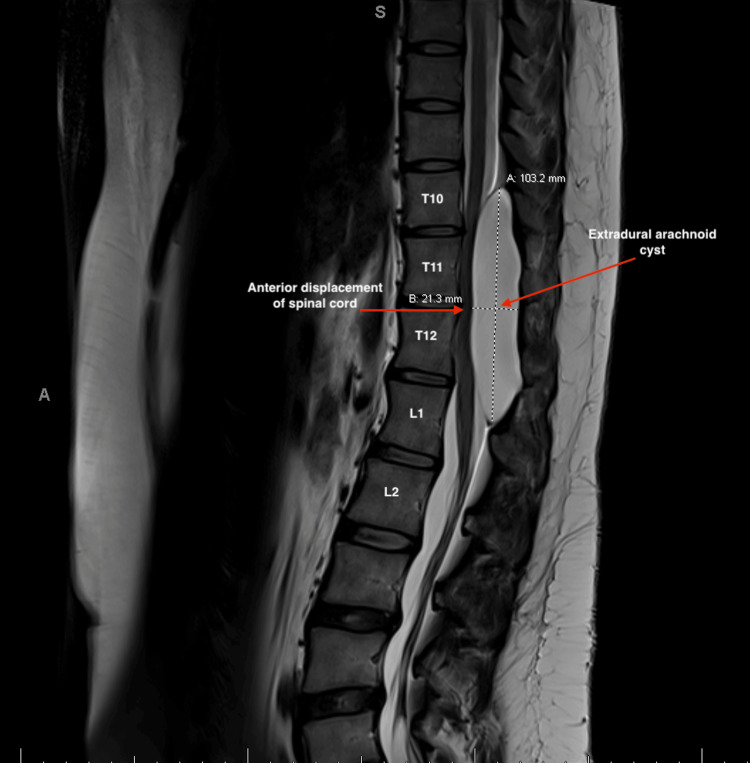
Preoperative sagittal T2-weighted MRI demonstrating the large, homogeneously hyperintense extradural cystic lesion at level of lower T10 extending to L1/L2. Anterior displacement of the spinal cord and posterior vertebral body scalloping are evident, consistent with a chronic process. Orientation label: S - superior; A - anterior

**Figure 2:**
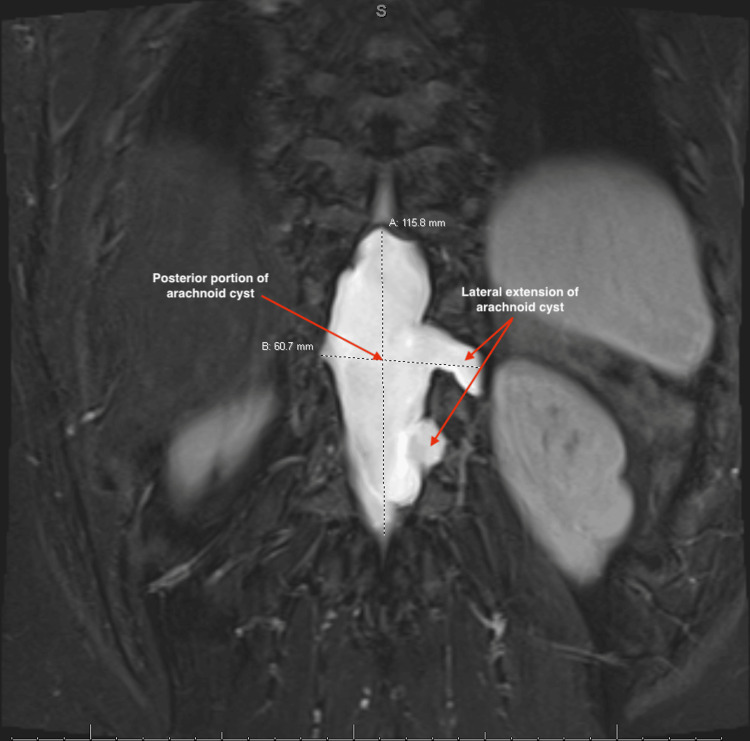
Preoperative coronal T2-weighted MRI showing the lobulated morphology of the cyst, measuring approximately 116 mm × 61 mm, rounded from MRI measurements, with lateral foraminal extension at the left T11/T12, T12/L1, and L1/L2 levels. Orientation label: S - superior

**Figure 3:**
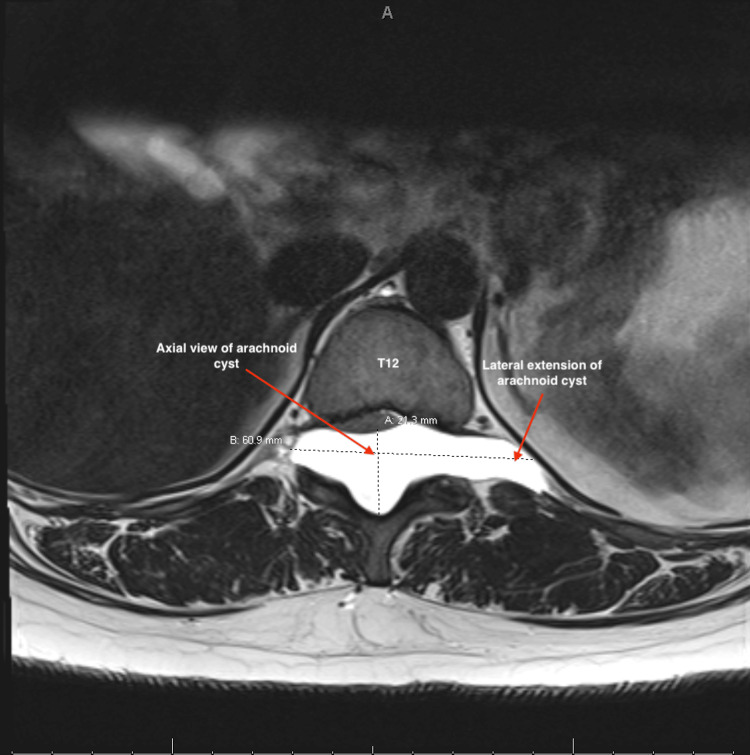
Preoperative axial T2-weighted MRI at the level of T12/L1 with maximum transverse extent approximately 61 mm, rounded from MRI measurements, demonstrating the extradural cystic lesion with anterior compression of the thecal sac. Orientation label: A - anterior

Surgical management

The patient underwent surgery under general anesthesia in the prone position with continuous intraoperative neurophysiological monitoring; a standard posterior midline approach was used. Partial laminectomy of the inferior half of T10, complete laminectomy of T11 to T12, and partial laminectomy of the superior half of L1 were performed. The facet joints were preserved to avoid future spinal instability, as the patient is young. Adequate surgical exposure of both the superior and inferior ends of the cyst was achieved.

Rostral-to-caudal microsurgical dissection was performed to separate the cyst wall from the underlying dura. The dissection was uncomplicated along the central and caudal extents of the cyst, which led to near-total excision of the main cyst body. However, at the rostral and left-lateral margin, the cyst was found to be multiloculated with a component that was adherent to the dura at the level of left T11/T12 neural foramen. The accessible, loosely adherent portions of this loculated segment were aspirated and excised. The densely adherent intra-foraminal component at the left T11/T12 level was fenestrated in order to preserve dural integrity, prevent CSF leakage, and avoid injury to the left T11/T12 nerve root. At the end, hemostasis was confirmed, and the wound was closed.

Histopathological analysis

The excised cyst tissue was submitted for histopathological analysis. Gross examination revealed a single fragment of soft, tan-colored cystic tissue measuring 4.0 × 1.5 × 0.4 cm, submitted in its entirety across three tissue cassettes. This gross specimen measurement represents the excised cyst wall and does not reflect the full preoperative radiological cyst size, as the adherent foraminal component was fenestrated rather than completely excised.

Hematoxylin and eosin (H&E) stained sections demonstrated pleated fibrovascular tissue lined by sparsely spaced, small, monomorphic arachnoid cells. Denuded areas of the cyst lining were evident in most regions. Foci of mineralization were also identified in the specimen. Immunohistochemical analysis with CD34 highlighted scattered vessels. The specimen was negative for malignancy. These histological features, along with radiological findings, are characteristic of a benign spinal arachnoid cyst.

Postoperative course, follow-up, and patient outcomes

Immediate postoperative MRI obtained on the first postoperative day demonstrated expected postoperative changes at the operative bed, including enhancing granulation tissue, and a small residual cystic component at the T11/12 disc level measuring 34.5 × 12.5 mm extending into the corresponding neural foramen (Figures 4-5). No new postoperative neurological deficit was observed.

**Figure 4:**
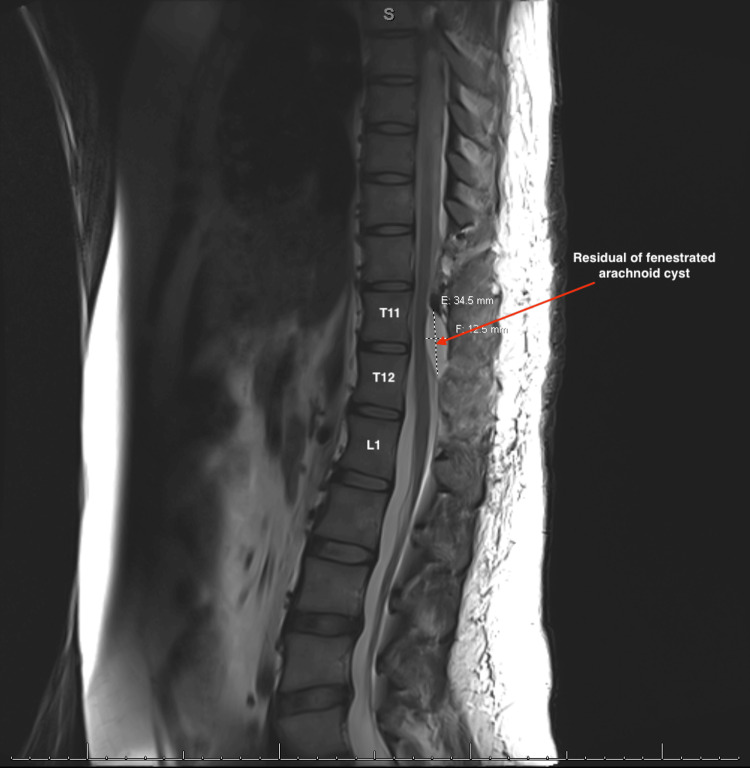
Immediate postoperative sagittal T2-weighted MRI (first postoperative day) demonstrating expected surgical changes at the operative bed with a small residual cystic component measuring 34.5 mm × 12.5 mm at the T11/12 level, corresponding to the selectively fenestrated intra-foraminal component at the left T11/T12 level. Orientation label: S - superior

**Figure 5:**
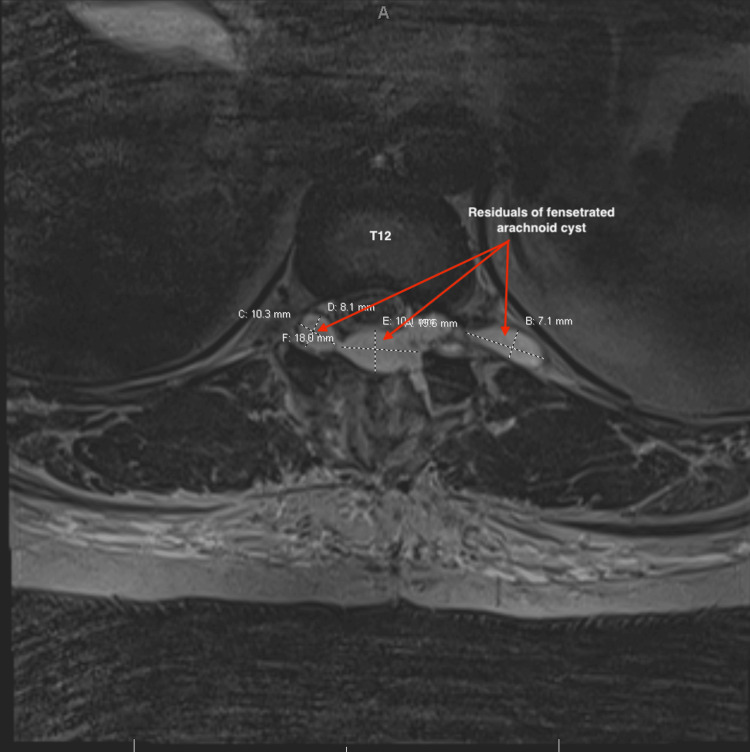
Immediate postoperative axial T2-weighted MRI (first day postoperative) demonstrating the fenestrated foraminal cystic components, with residual measurements of 7.1–18.0 mm, representing separate residual fenestrated loculated components. Orientation label: A - anterior

The final follow-up MRI at 10 months postoperatively confirmed complete resolution of all extradural arachnoid cystic lesions (Figures 6-7). Ectasia of the thecal sac and prominence of the subarachnoid spaces in the T10 up to T12 level were consistent with postoperative decompression. No recurrent compressive extradural cystic lesion or postoperative deformity was identified on follow-up MRI.

**Figure 6:**
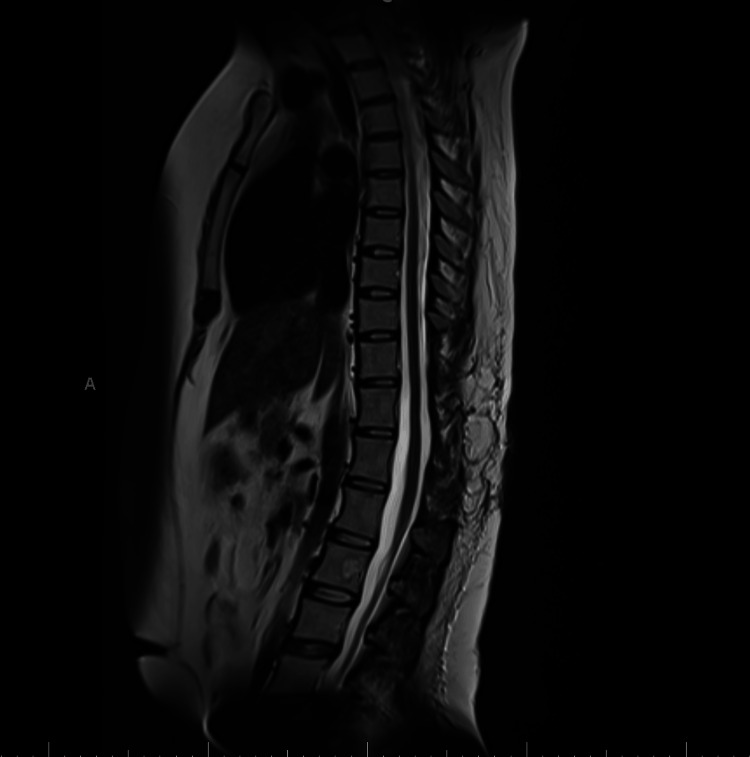
Final follow-up sagittal T2-weighted MRI demonstrating complete decompression of the spinal canal with no residual extradural arachnoid cystic lesion. Laminectomy changes are visible at T10–T12. Persistent posterior vertebral body scalloping reflects chronic pre-existing bony remodeling. Orientation label: A - anterior

**Figure 7:**
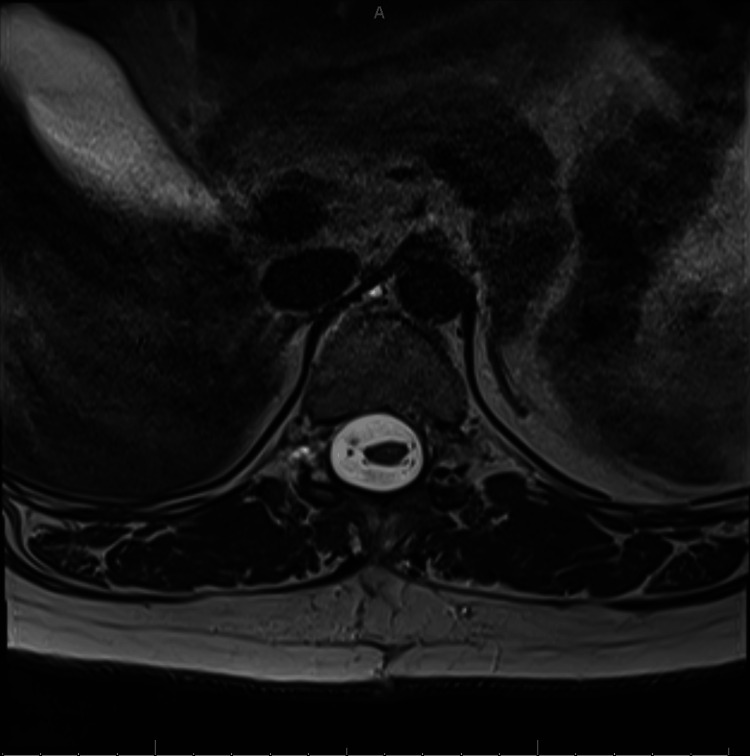
Final follow-up axial T2-weighted MRI at T12-L1 level, confirming complete resolution of the extradural arachnoid cystic lesion. Ectasia of the thecal sac is consistent with post-decompression changes. Orientation label: A - anterior

Clinically, the patient demonstrated significant functional recovery. Pain scores were obtained from the patient using the 0-10 numeric rating scale, improved from 9/10 preoperatively to 4/10 at 10-month follow-up a level tolerable without analgesic medication. Bilateral lower extremity, weakness, paresthesia and urinary incontinence were reported as fully resolved by the patient.

## Discussion

Spinal extradural arachnoid cysts predominantly involve the thoracic and thoracolumbar spine [2-4,7]. The present case has several atypical features that represent challenging surgical management: the cyst was large, extending across the thoracolumbar junction from lower T10 to upper L2, was multiloculated, and demonstrated intra-foraminal extension at multiple left-sided levels. Although foraminal extension has been described in articles, the combination of those features and adherent multilevel foraminal involvement made this case surgically challenging [4,13,14].

The posterior vertebral body scalloping and foraminal widening identified on preoperative MRI in this patient are hallmark imaging features of long-standing arachnoid cysts, attributable to the chronic, sustained mechanical pressure of the slowly expanding lesion on adjacent structures [2,4,13,14]. This combination of findings is characteristic of a benign, chronic, slow-growing process and is radiologically different from more rapidly progressive or malignant spinal neoplasms. The absence of post-contrast enhancement provided additional support for a non-neoplastic etiology, consistent with the Nabors t0ype IA classification [1,2,4,14].

Histopathological examination confirmed the diagnosis, demonstrating the characteristic of spinal arachnoid cysts: fibrovascular tissue lined by sparsely spaced, small, monomorphic arachnoid cells [1,3,12,14]. CD34 immunostaining highlighted scattered stromal vessels. The specimen was negative for malignancy, with no evidence of neoplasia.

In this case, the main surgical decision was whether to pursue complete removal of the foraminal component or to fenestrate it safely because of its adherence to the dura and nearby nerve root. The optimal surgical treatment for spinal extradural arachnoid cysts remains controversial, although many surgeons prefer complete excision when feasible [7,9,11,13]. However, when the cyst wall is densely adherent to the dura or nerve root, as was encountered in this case at the level of left T11/T12 foramen, aggressive attempts at complete excision may carry a risk of dural injury, CSF leakage, nerve root injury, and potential complications related to wider exposure, including postoperative deformity or instability [7,9,10]. Fenestration and decompression of the adherent residual loculation were chosen to reduce cyst pressure while avoiding dural or nerve root injury. Similar limited surgical approaches have been reported as a safe and effective strategy in this specific operative scenario, particularly when complete cyst excision may increase postoperative risk [7,9,11,12]. The complete MRI resolution of the residual foraminal component on postoperative imaging in this case strongly supports this rationale and suggests close MRI surveillance rather than early re-intervention.

The clinical recovery documented in this patient, including pain score improvement from 9/10 to 4/10 and complete resolution of weakness, bilateral paresthesia, and urinary incontinence, is consistent with published series reporting favorable neurological outcomes following SEAC surgery. In these studies, postoperative recovery has been associated with factors such as symptom duration and severity or extent of preoperative neural compression [7,10,13].

Finally, the decision to perform near total resection of spinal extradural arachnoid cyst with fenestration of densely adherent intraforaminal component while preserving facet joints, is done to achieve normal neurological function with the least possible long-term complication. Facet preservation during multilevel laminectomy is critical to preventing postoperative junctional kyphosis, a complication of particular long-term significance in a young patient [7,9,10]. No recurrent compressive extradural arachnoid cystic lesion or postoperative deformity was identified on follow-up MRI, supporting this conservative approach to posterior column preservation in the present case. However, as this is a single case with a 10-month follow-up, longer follow-up and larger case series are needed to better assess recurrence risk and long-term outcomes.

## Conclusions

We report the successful surgical management of a large thoracolumbar spinal extradural arachnoid cyst (Nabors type IA) via decompressive laminectomy, near-total cyst resection, and preservation of facet joints to avoid future complications, with histopathological confirmation of a benign arachnoid cyst negative for malignancy. In cases where the intra-foraminal cyst component is densely adherent to the dural sleeve or foraminal neural structures, selective fenestration may be considered over attempted complete excision. This approach preserves dural integrity, lowers the risk of cerebrospinal fluid leak, and avoids unnecessarily wide bony resection. Significant functional recovery was achieved, with pain improving from 9/10 to 4/10 at 10-month follow-up (tolerable without analgesics), complete resolution of weakness, paresthesia, and bladder function. The residual fenestrated intraforaminal component was no longer seen on the 10-month follow-up MRI.
